# A Disruption in the Balance of Attentional Systems Plays a Role in Trait Anxiety

**DOI:** 10.3390/brainsci10100761

**Published:** 2020-10-21

**Authors:** Mark R. Minnick, Koraly E. Pérez-Edgar, José A. Soto

**Affiliations:** Department of Psychology, The Pennsylvania State University, University Park, PA 16801, USA; marmin3@gmail.com (M.R.M.); kxp24@psu.edu (K.E.P.-E.)

**Keywords:** trait anxiety, attentional control, visual detection, attention bias, skin conductance

## Abstract

Elevated levels of anxiety are associated with attentional threat biases and inefficient attentional control, with the latter requiring sustained cognitive effort. The current study assessed self-reported and behavioral evidence of attentional functioning, along with electrodermal activity (EDA; measured via changes in skin conductance level [SCL reactivity]) as an index of sympathetic arousal, to examine whether these vulnerabilities are evident among individuals with elevated trait anxiety (non-clinical). Fifty-nine participants completed a working memory span task measuring attentional control under high cognitive load. A visual change detection task assessed stimulus-driven attention as an indicator of vigilance to non-threatening visual information. Trait anxiety was self-reported. SCL was captured at rest and during the working memory task. Results revealed that trait anxiety was positively related to speed of visual change detection, without accuracy costs, suggesting enhanced vigilance for neutral visual information among those higher in trait anxiety. Trait anxiety also moderated the relation between change detection speed and attentional control, such that attentional vigilance was not associated with variation in attentional control for individuals higher in trait anxiety. However, for individuals lower in trait anxiety, vigilance was negatively associated with attention control. The relationship between vigilance and attentional control was also moderated by SCL reactivity such that the association was only significant at lower SCL reactivity levels. Taken together, results suggest that individuals higher in trait anxiety demonstrate greater attentional control in the service of visual detection, but greater attentional control may come at the cost of increased sympathetic arousal.

## 1. Introduction

Individuals with elevated anxiety display attentional threat biases that may impact cognitive performance. Attentional control theory (ACT) [1] proposes that the experience of anxiety is marked by a disrupted balance between two components of attention: an attentional system driven by salient stimuli and an associated attentional system necessary for goal-directed control over cognition and behavior. Enhanced stimulus-driven attention, as evidenced by attentional biases to threat, may come at the cost of efficiency in goal-directed attentional functioning. This disruption then requires a significant investment of compensatory cognitive effort in order maintain effective attentional control [1,2].

Recent work [3,4] suggests that inefficient attentional control associated with elevated trait anxiety (as differentiated from individuals with clinical anxiety symptoms) is evident even in the absence of threatening stimuli, and thus may be indicative of a trait-level information processing style that confers vulnerability to developing clinically-salient anxiety symptoms [3]. The current study explores patterns of attentional functioning associated with variation in trait anxiety by taking a novel multimethod (behavioral, psychophysiological, and self-report) approach. Specifically, we examine how the balance between stimulus-driven and goal-directed attention on tasks that do not present threatening stimuli varies with self-reported levels of trait anxiety. We also explore whether the theorized deployment of compensatory effort to maintain attentional control may be evident in an index of sympathetic arousal (i.e., skin conductance level).

### 1.1. Attentional Functioning in Trait Anxiety

High levels of trait anxiety can be deleterious to emotional well-being and contribute to deficits in daily functioning, due in part to associated cognitive difficulties [1,5]. A key feature of trait anxiety is an attentional bias for detecting, and a difficulty disengaging from, threatening information (see [6] for a review). Such biases can be to threatening external environmental cues [7] or even threatening internal cues such as worry [8] or perceived somatic reactivity [9]. Ample evidence suggests that these biases are an important etiological factor [10] likely modulating the influences of temperamental vulnerability and the environment in increasing the risk that trait anxiety leads to functionally-impairing anxiety symptoms [11].

Attention is supported by separate but interactive neurological networks, including a goal-driven network necessary for directing attention in a top–down fashion and a stimulus-driven network influenced by the salience or relevance of environmental stimuli [12]. The presence of attentional biases represents an increased allotment of stimulus-driven attentional resources in order to maximize the likelihood of detecting threat [1]. This attentional bias often results in a distorted perception of threat in the environment among individuals with higher levels of trait anxiety, with even ambiguous or neutral stimuli being interpreted as threatening [8,13]. ACT posits that allotting attentional resources so broadly for threat monitoring comes at the cost of available resources for goal-directed attentional functioning, or attentional control. Limited attentional control can impair performance on complex cognitive tasks [14] and effective regulation of negative emotional experiences [15,16].

Trait anxiety is associated with self-reported deficits in attentional control [17]. According to ACT, behavioral evidence of anxiety-related attentional control deficits should be evident in tasks relying on the central executive system, as illustrated in Baddeley’s working memory model [14,18]. The central executive system is responsible for attentional control by supporting effective attention shifting between tasks, inhibiting the influence of distracting stimuli on task performance, and monitoring and updating information in working memory [14,19]. Typically, measures of performance focus on accuracy. However, below the surface, performance also reflects the speed and the level of effort needed to be accurate. While increased stimulus-driven attention among individuals with elevated levels of trait anxiety should limit resources necessary for central executive recruitment, enhanced cognitive effort allows for maintenance of effective (i.e., accurate) performance on tasks requiring attentional control [1,20]. However, the influence of increased stimulus-driven attention on cognitive resource availability should limit the efficiency (response latency) of attentional control performance [1,2]. These costs should be further modulated by state anxiety (anxiety experienced in the moment), the presence of threat-related stimuli, and/or increased cognitive load [1].

### 1.2. Representative Evidence of Imbalanced Attentional Functioning

Attentional biases delay disengagement when processing threatening stimuli or when performing under conditions of stress or heightened state anxiety [21,22,23,24]. In addition, the predicted attentional control difficulties associated with trait anxiety are evident in both the behavioral and neural data, even in the absence of state anxiety inductions and threat-related stimuli. Studies involving both flanker [4] and antisaccade [25] tasks have found that higher levels of trait anxiety predicts deficits in the efficiency of central executive recruitment needed to inhibit interference by neutral or incongruent stimuli. Increased frontal and prefrontal activation on a task requiring the maintenance and manipulation of working memory was associated with higher trait anxiety in spite of equivalent behavioral performance [26]. Consistent with ACT, activation in the absence of a cost to performance might indicate enhanced effort in the service of maintaining attentional control [26].

Although the above findings provide robust evidence for trait anxiety-related costs to attentional control, direct evidence of increased stimulus-driven attention is not well established. The available findings are largely limited to evidence of faster threat detection with increases in trait anxiety [27]. Trait anxiety is expected to include hypervigilant monitoring for threat [28]. As such, individuals with higher levels of trait anxiety should exhibit enhanced processing of environmental stimuli regardless of the presence of threat [29], as they select and label stimuli as threat or non-threat. Indeed, individuals with high trait anxiety, but not low trait anxiety, process non-salient visual distractors even while engaged in a task placing high demand on attentional control [30], displaying faster detection of visual stimuli located on the periphery of primary stimuli in a letter search task [29], and enhanced early visual information processing for neutral information via event-related potentials (ERPs) [31]. However, it is difficult to determine whether trait anxiety is associated with an isolated benefit to early information processing or whether the demonstrated benefits manifest only when central executive resources are engaged. As such, we need to more directly test the presence of trait anxiety-related advantages in early information processing.

Additionally, more work is needed to clarify the relation between trait anxiety level and central executive functioning. In particular, research on working memory updating deficits is limited, and the findings have largely failed to demonstrate efficiency costs as typically measured (i.e., response time costs; [2]). According to Eysenck and colleagues [1], such findings are to be expected as individuals with elevated anxiety will demonstrate less efficiency costs on tasks relying on updating functioning—such as complex working memory span tasks versus tasks relying on inhibition and shifting—except, of course, under high stress. Updating relies primarily on transient memory storage rather than attentional control [19,32], although others argue that attentional control is an important component of working memory updating [33,34]. Consequently, updating should be influenced by trait anxiety, even if costs to updating efficiency are not as significant as inhibition and shifting costs. In fact, some work (e.g., [35]) indicates that trait anxiety is associated with enhanced neural activity during conflict monitoring, an ability thought to be closely related to working memory updating [2].

Also limited in the extant literature is evidence about how the cognitive effort necessary for trait-anxious individuals to maintain effective attentional control manifests outside of task performance. The effort of individuals high on trait anxiety in response to attentional control demands is associated with enhanced frontal activity [1,2]. However, converging evidence suggests that a need to deploy enhanced effort may additionally contribute to peripheral nervous system activity (i.e., sympathetic and parasympathetic nervous system activation) that could contribute to the subjective experience of state anxiety. Cognitive effort in response to decision-making tasks and to increasing demands on attentional control lead to sympathetic arousal (as measured via heart rate and electrodermal activity) in normative samples [36,37]. Such autonomic activity can, in general, also contribute to negative emotional experience [38]. Further, perceived somatic responses associated with increased sympathetic arousal (such as increased heart rate) are often experienced as sources of threat by trait-anxious individuals [9]. Given the potential importance of autonomic reactivity to the experience of cognitive effort in trait-anxious individuals, the current study utilized electrodermal activity as a psychophysiological index of arousal associated with task performance.

### 1.3. Current Study

A disrupted balance in attentional processes may be indicative of a trait anxiety-related information processing style [3]. However, there is limited research testing the predictions of ACT in individuals with variation in trait anxiety without inducing state anxiety and without utilizing threat-related stimuli. Demonstrating that the predictions of ACT hold in this context would further support the notion that an imbalance in the influence of attentional systems on cognitive performance is associated with variation in levels of trait anxiety that do not reach clinical concerns. Along with directly testing the interrelations of trait anxiety level with the effectiveness and efficiency of working memory updating functioning, we took a novel approach to testing whether trait anxiety predicts the relation between goal-directed attentional performance—as indicated by updating functioning—and early visual information processing.

We used a one-shot change detection task [39] to test for patterns of early visual information processing indicative of a general hypervigilance to visual information and increased stimulus-driven attention. We employed an Operation Span task (O-Span; e.g., [40]) to test updating functioning under a high concurrent processing demand. Electrodermal activity data were simultaneously collected to determine whether the predicted enhanced deployment of cognitive effort is reflected in sympathetic arousal [37,41]. Finally, given that self-report measures have been valuable in examining anxiety-related attentional phenomena in prior work [42], we were also interested in the extent to which self-reported attentional functioning is associated with trait anxiety, and whether such self-report data are consistent with behavioral measures.

### 1.4. Hypotheses

(1) Higher levels of trait anxiety will be associated with enhanced early visual information processing, as indicated by a shorter change detection response time without an accompanying cost to accuracy. This pattern is in line with the ACT model’s prediction that accuracy is typically preserved among anxious individual completing a moderately difficult task. However, to do so, individuals must slow down or expend great energy—both of which can be considered a marker of poor efficiency.
(2) Consistent with prior behavioral findings (see [1]), we did not expect trait anxiety to directly predict either efficiency or effectiveness of working memory updating performance on the O-Span task. Trait anxiety will moderate the relation between early visual information processing and effectiveness of updating functioning for individuals higher in trait anxiety. The relation between the change detection response time and performance effectiveness on the O-Span task would be weaker.
(3) We expected that trait anxiety would be positively related to SCL reactivity (i.e., change in SCL from baseline to trial) during the O-Span, reflecting increased effort. We also predicted that SCL reactivity would similarly moderate the relation between the change detection response time and O-Span recall, while controlling for the influence of trait anxiety level.
(4) Finally, to test the importance of perceived traits, we examined the relation of perceived attention control and temperament (i.e., negative affect and extraversion/surgency [43,44]) with trait anxiety levels. We expected that trait anxiety would be associated with greater negative affect, lower extraversion/surgency, lower attentional control, and greater neutral perceptual sensitivity.

## 2. Materials and Methods

### 2.1. Participants

Fifty-nine undergraduate participants were recruited from a large public university in the early phase of a larger, quasi-experimental study of the relations between trait anxiety and a wide range of executive functions. The trait anxiety level of the sample in the current study (State Trait Anxiety Inventory; STAI—Form Y-2; [45]) was at a normative level and normally distributed (*n* = 58, *M [mean]* = 37.55, *SD* [standard deviation] = 9.59, range = 21 to 64; one participant was eliminated due to an error in collecting trait anxiety self-report). Participants identified as primarily female (63.8%) and Caucasian (87.9%) and were between ages 18 and 22 (*M* = 18.96, *SD* = 0.92).

### 2.2. Apparatus

#### 2.2.1. Audiovisual

Participants were presented with the experimental materials and all procedural elements on a 33-by-51 cm computer monitor. The PsychData^®^ (Psychdata, State College, PA, USA) web-based survey utility was used for presenting self-report scales, and E-Prime 2.0^©^ (Psychology Software Tools, Inc., Pittsburgh, PA, USA) software was used for the presentation of behavioral tasks.

#### 2.2.2. Physiology

A Biopac^©^ MP150 (Biopac Systems, Inc., Goleta, CA, USA) device was used to capture physiological data. The device is composed of a microcomputer and an eight-channel polygraph. Although only electrodermal activity (i.e., SCL) data were utilized in the current study, cardiac impedance, electrocardiography, peripheral pulse, and skin temperature data were also collected. SCL was collected with self-adhering electrodes placed on the index and ring fingers, at the middle phalanx of each. The peripheral pulse, SCL, and skin temperature were collected from the non-dominant hand, so as to not interfere with task completion. Physiological data were collected with AcqKnowledge^©^ software (Biopac Systems, Inc., Goleta, CA, USA), and the data were processed using Mindware^©^ software (Mindware Technologies LTD, Westerville, OH, USA). The data processing software computed the average skin conductance amplitude (in microsiemens) across a given epoch in the trials of interest (e.g., baseline period, during segments of the attentions task) which corresponds to the average tonic level of skin conductance reactivity. Importantly, skin conductance level as measured here does not take into account the number of individual skin conductance responses (i.e., Galvanic Skin Responses) over the same time window, although the overall tonic level of skin conductance and the number of skin conductance responses are related and both are reliable indicators of sympathetic arousal even though they more accurately reflect a balance between sympathetic and parasympathetic activity.

### 2.3. Measures

#### 2.3.1. Trait Anxiety Level

Anxiety level was assessed using the STAI—Form Y [45], a 40-item questionnaire including 20 items assessing trait anxiety level and 20 items assessing state anxiety level. The trait scale asks participants to what extent certain experiences (e.g., “I feel nervous and restless”) apply to them generally. Items are rated on a 4-point Likert-type scale (“1 = Almost never,” “4 = Almost always”). Summed item scores in each scale yield possible total scores of 20–80. The STAI has demonstrated good reliability and validity in assessing individuals for anxiety level in both clinical and research contexts [45]. The STAI demonstrated excellent reliability for this sample (α = 0.95).

#### 2.3.2. Dispositional Attentional and Socioemotional Functioning

The Adult Temperament Questionnaire (ATQ) short form [46] is a 77-item questionnaire measuring four higher-order temperament factors—negative affect, extraversion/surgency, effortful control, and orienting sensitivity. Items are rated on a 7-point Likert-type scale from “1—Extremely untrue of you” to “7—Extremely true of you.” Scales within each higher-order factor assess the following: negative affect factor scale—fear, sadness, discomfort, and frustration scales; extraversion/surgency factor scale—sociability, positive affect, and high intensity pleasure scales; effortful control factor scale—attentional control, inhibitory control, and activation control scales; orienting sensitivity factor scale—neutral perceptual sensitivity, affective perceptual sensitivity, and associative sensitivity scales (for descriptions of all scales, see [46]).

Scores are calculated from the mean of the items comprising each scale/factor. Factors demonstrated acceptable reliability for our sample (α = 0.76 to 0.81). We used negative affect and extraversion/surgency to capture dispositional foundations of trait anxiety such as proneness towards behavioral inhibition and internalizing problems [43]. In order to capture dispositional goal-directed attentional functioning, we utilized the attentional control scale, which assesses one’s ability to focus and shift attention. Dispositional stimulus-driven attention was assessed with the neutral perceptual sensitivity scale, which captures sensitivity to detect low-intensity internal and external stimuli [46].

#### 2.3.3. Visual Change Detection Task

The task presents participants with a sample array of equally shaped colored squares for a short duration (200 ms). Following a brief delay (1000 ms), participants saw a test array that was either identical to the sample array or had one square changed in color. Participants were to respond as quickly and accurately as possible as to whether the arrays were identical (indicated with the left mouse button) or included a change (indicated with the right mouse button). The test array remained on screen until the participant responded. Arrays varied in size, containing four, seven, or ten squares, with 40 trials of each array size (120 total trials). In half of the trials of each array size, the test array was identical to the exposure array, and in the other half, one of the squares changed color. Trials were administered in random order. Because of the low processing demand associated with the task and the brief sample array encoding time allotted to participants, we believe the response time in this task provides an index of early visual information processing ability that could indicate an influence of stimulus-driven attention [39,47].

#### 2.3.4. Operation Span Task

The O-Span task measures the central executive’s updating function, requiring participants to retain a series of words in working memory while evaluating the accuracy of mathematical equations [40,48]. Complex working memory span tasks such as the O-Span have demonstrated good reliability and validity as measures of working memory capacity in the context of high processing demand [49]. Participants saw a series of equation and word sequences. First, participants were given 3750 ms to respond to the accuracy of an equation (e.g., “6/3 + 4 = 6”; “1 + 3 × 2 = 9”). “Correct” responses were indicated with a left mouse button press and “incorrect” responses were indicated with a right mouse button press. A 500 ms blank screen was then presented, followed immediately by a word to be remembered, which remained on the screen for 1250 ms. Subsequently, another equation was presented, followed by another word to be remembered, and so on.

Equation and word sequences were presented in sets with between two and six equation–word pairs per sets. The sets were presented in random order. At the end of each set, participants were instructed to write on a provided worksheet the words which appeared in the set. To encourage participants to attend to the processing-demanding equation trials, and not simply rehearse the words, instructions indicated the importance of maintaining a high level of accuracy on the equation judgments. The task contained 15 blocks of equation–word pairs, 3 of each set size, for a total of 60 trials. An O-Span retention score was calculated from the number of words recalled across all sets, with a maximum score of 60. Working memory updating effectiveness was operationalized as the O-Span retention score, and the response time to the equation decisions was utilized as an index of efficiency.

#### 2.3.5. SCL Reactivity

SCL was collected before and during the O-Span task. A two-minute baseline period prior to task instructions was extracted. Given the length of the O-Span task (6 min), we extracted SCL data in three two-minute epochs (as the average tonic level across each epoch) starting from the outset of the first block of trials in order to identify whether a relation between trait anxiety and SCL reactivity was consistent over time. SCL Reactivity was calculated by subtracting the mean SCL of the two-minute baseline from the mean SCL of each of the task epochs.

### 2.4. Procedure

Participants were seated approximately three feet from the computer monitor, and first completed a series of self-report measures, including the STAI and the ATQ. A research assistant then placed the physiological data collection sensors on the participants. The change detection and O-Span tasks were then presented, counterbalanced for order.

### 2.5. Data Reduction and Assumption Checks

A significant amount of SCL data were unusable, accounting for the reduced sample sizes in the analyses reported below. Unusable SCL data were typically associated with EDA signals not being of sufficient strength to be detected by sensors, possibly due to a number of factors outside of our control including the pigmentation level of the skin and skin moisture level. The outlier labeling rule [50] was used to screen outliers in each participant’s response time data for the O-Span and each change detection task condition (see Table 1 for descriptive statistics). All compiled predictor and criterion variables were additionally screened for normality. Outlier removal excluded approximately 2% of collected data. The SPSS PROCESS bootstrapping macro [51] was used to test moderation models. For each analysis, PROCESS was configured for 1000 bootstrap samples in order to mean center variables prior to creating interaction terms and to estimate simple slopes at the mean, +1 standard deviation, and −1 standard deviation of the predicted moderator. The Mahalanobis distance was calculated for each multivariate analysis to screen for any multivariate outliers (based on a χ^2^ distribution at *p* < 0.001; e.g., [52]). One participant was classified as a multivariate outlier for analyses involving ATQ data and was excluded from analysis, and a data collection error resulting in missing ATQ data excluded another participant.

## 3. Results

### 3.1. Early Visual Information Processing

Within-subjects ANOVAs demonstrated an effect of change detection array size on both response accuracy (*F*(2, 108) = 115.91, *p* < 0.001, η_p_^2^ = 0.68) and reaction time (*F*(1.80, 93.61) = 22.83, *p* < 0.001, η_p_^2^ = 0.30) indicating costs of increased perceptual load. Bonferroni-corrected post-hoc analyses indicated that change detection accuracy decreased with each increase in array size (all *ps* < 0.001). Reaction time increased from the small array trials to the medium array trials (*p* < 0.001), but only marginally increased from the medium to the large array trials (*p* = 0.066). Dependent t-tests revealed an effect of change condition on accuracy across all array sizes (all *t*s > 8.29; all *p*s < 0.001).

To test whether this discrepant performance might indicate a biased response style due to difficulty detecting changes, we compared the zero-order correlations between accuracy on change trials and accuracy on non-change trials for each array size. There was no association between accuracies on the small (*p* = 0.69) and medium (*p* = 0.76) array trials. However, there was a significant negative correlation on large array trials (*r*(55) = −0.34, *p* = 0.01), suggesting an inability to detect changes on large array trials and a bias towards “no change” responding on those trials. For this reason, large array trials were removed from further analyses.

Within-subjects ANOVAs were re-run including only the small and medium arrays, with trait anxiety score included as a covariate. Trait anxiety did not moderate the effect of array size on either accuracy (*F*(1, 53) = 0.16, *p* = 0.69) or reaction time (*F*(1, 51) = 0.17, *p* = 0.68). Consistent with our prediction, trait anxiety predicted the response time across conditions (*F*(1, 51) = 4.64, *p* = 0.036, η_p_^2^ = 0.08) and was not predictive of change detection accuracy across array sizes (*F*(1,53) = 0.91, *p* = 0.34). Although the negative relation between trait anxiety level and the response time was strong for small arrays (*r*(53) = −0.32, *p* = 0.019), but not for medium arrays (*r*(55) = −0.28, *p* = 0.079), these correlations were not significantly different from each other (Z = −1.14, *p* = 0.252).

### 3.2. Balance between Early Visual Information Processing and Effectiveness of Working Memory Updating

**Figure 1:**
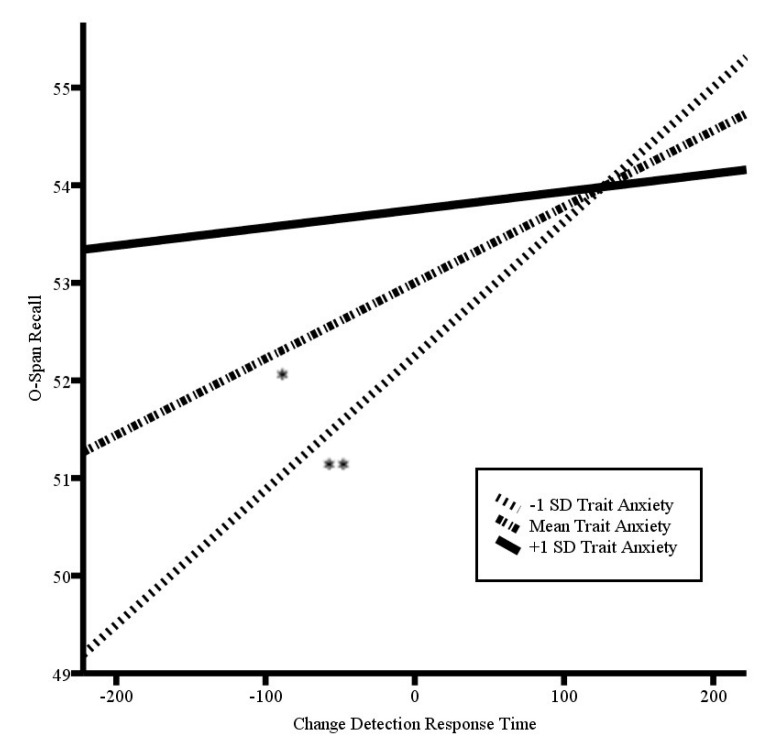
Plot of simple slopes of the centered change detection response time by trait anxiety level in predicting O-Span recall; ** *p* < 0.001; * *p* < 0.05. Higher scores on the O-Span recall represent greater attentional control. Higher scores in change detection response time indicate slower response time relative to the average detection speed (zero) and, therefore, less vigilance.

Zero-order correlations indicated that trait anxiety was not related to updating effectiveness (O-Span word recall) (*r*(52) = 0.06, *p* = 0.659). In addition there was no relation between trait anxiety and performance on the concurrent equation judgment task, in terms of either accuracy (*r*(49) = 0.12, *p* = 0.40) or reaction time (*r*(52) = −0.10, *p* = 0.502). The model testing the moderation of the relation between early visual information processing and effectiveness of working memory updating by trait anxiety (see Table 2) was significant (*F*(3,48) = 5.74, *p* = 0.002, *R*^2^ = 0.18). Trait anxiety level was not predictive of O-Span recall, *p* = 0.11. Consistent with our prediction, there was a significant interaction effect between trait anxiety and the change detection reaction time in predicting O-Span recall (*b* = −0.001, *p* = 0.002). At mean levels of trait anxiety, faster reaction time on the change detection task was predictive of lower O-Span recall (*b* = 0.008, *p* = 0.038). The relation was stronger at lower levels of trait anxiety (*b* = 0.01, *p* < 0.001). At higher trait anxiety levels, there was no relation between the change detection reaction time and O-Span recall (*b* = 0.002, *p* = 0.668). See Figure 1 for a plot of the simple slopes. These findings suggest that enhanced dispositional early visual information processing associated with trait anxiety does not come at the cost of effectiveness of updating performance.

### 3.3. Effortful Central Executive Recruitment as Indicated by SCL Reactivity

**Figure 2:**
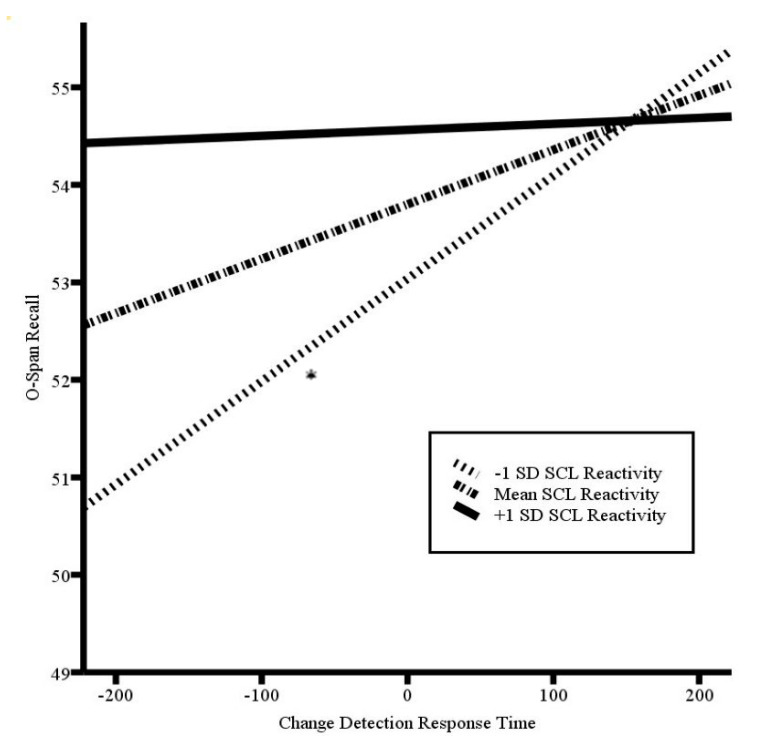
Plot of simple slopes of the centered change detection response time by skin conductance level (SCL) reactivity in predicting O-Span recall, controlling for trait anxiety level; * *p* < 0.05. Higher scores on the O-Span recall represent greater attentional control. Higher scores in change detection response time indicate slower response time relative to the average detection speed (zero) and, therefore, less vigilance.

Increased trait anxiety was related to greater SCL reactivity during the first two-minute epoch of the O-Span task (*r*(34) = 0.354, *p* = 0.04), but not during the second (*r*(34) = 0.320, *p* = 0.065) or third (*r*(34) = 0.29, *p* = 0.098) epochs, or as averaged across all three epochs (*r*(34) = 0.32, *p* = 0.058). As such, we re-ran the model predicting O-Span recall from the change detection reaction time, using SCL reactivity during the first. The overall model was significant (*F*(4, 29) = 3.94, *p* = 0.011, *R*^2^ = 0.17) (see Table 3). Consistent with our prediction, initial SCL reactivity moderated the relation between the change detection reaction time and O-Span recall (*b* = −0.000, *p* = 0.044). While lower SCL reactivity was associated with a negative relation between the change detection response time and O-Span recall (*b* = 0.01, *p* = 0.034), the relation was not significant at mean SCL reactivity (*p* = 0.326) or higher levels of SCL reactivity (*p* = 0.932). See Figure 2 for a plot of the simple slopes. These results suggest that enhanced effort early in the O-Span task, as indexed by SCL, moderated the balance between early visual information processing and updating-reliant attentional control over and above the influence of trait anxiety.

### 3.4. Perceived Attentional Functioning

Table 4 presents zero-order correlations between trait anxiety and ATQ variables of interest. Trait anxiety was positively related to negative affect (*p* < 0.001) and negatively related to extraversion/surgency (*p* < 0.001). Trait anxiety was negatively related to attentional control (*p* = 0.011), but was not significantly related to neutral perceptual sensitivity (*p* = 0.093). Neutral perceptual sensitivity and attentional control were not related with each other (*p* = 0.473), and thus likely capture independent dispositional attentional constructs in this sample. Additionally, extraversion/surgency and negative affect were also not related with each other (*p* = 0.121).

A hierarchical multiple regression was conducted to test the predictive value of perceived dispositional attentional functioning in trait anxiety score, over and above dispositional socioemotional functioning. Available demographic variables—gender, ethnicity, and age—were controlled for in the first step of the regression and did not significantly contribute to the model (*R*^2^ = 0.02, *p* = 0.744). Adding extraversion/surgency and negative affect to the model explained an additional 29.2% of the variance (*p* = 0.002). Both extraversion/surgency (β = −0.37, *p* = 0.006) and negative affect (β = 0.37, *p* = 0.008) were significantly related to trait anxiety prior to including attentional factors. Consistent with our predictions, the attentional scales contributed significantly to the model (*p* = 0.009), explaining an additional 12.1% of the variance, and each was significantly related to trait anxiety. Attentional control was negatively related to trait anxiety (β = −0.35, *p* = 0.008). Contrary to expectations, neutral perceptual sensitivity was negatively related to trait anxiety (β = −0.25, *p* =.037). The contribution of extraversion/surgency increased in the full model (β = −0.45, *p* < 0.001). Counter to our prediction, after including the attentional variables, negative affect no longer significantly contributed to the model (*p* = 0.253). These results suggest that trait anxiety can be predicted from both perceived traits and observed performance (e.g., perceived attention control and attention task behavior).

## 4. Discussion

The current study provided a novel demonstration that the relation between early visual information processing and updating performance varies as a function of trait anxiety levels. The current study disambiguated the components of performance by separately examining accuracy, speed, and effort. Results indicate that, unlike individuals lower in trait anxiety, individuals higher in trait anxiety can maintain attentional control under high cognitive load even when they display vigilance to environmental cues, marked by enhanced processing of visual information. The current findings also suggest that this trait anxiety-dependent pattern between early information processing and attentional control may carry over across time and task (e.g., [29]).

On the surface, an enhanced ability to quickly detect environmental stimuli without a noteworthy cost to attentional control could be interpreted as a functional advantage. However, the need to rely on enhanced effort to combat the influence of stimulus-driven attention and maintain attentional control may, itself, be problematic as it negatively impacts efficiency and effectiveness over time. Our findings suggest that the effort deployed in maintaining working memory updating performance during the O-Span task may increase sympathetic nervous system reactivity. Such reactivity could be distressing for individuals higher on trait anxiety given their attentional biases towards somatic sources of threat [9]. The presence of greater early information processing among individuals higher on trait anxiety, regardless of attentional control ability, is consistent with the notion that trait anxiety is marked by a hypervigilance for environmental sources of threat [28].

For individuals higher in trait anxiety, enhanced resource engagement for threat monitoring may not depend on the amount of resources concurrently engaged in attentional control, as found by Berggren and colleagues [29], or on dispositional attentional control ability, as the current data suggest. On the other hand, individuals lower in trait anxiety are not prone to enhanced vigilance towards distracting information while under high concurrent cognitive load (e.g., [30]) or, as indicated in the current findings, when they simply have better dispositional attentional control. An important conclusion to be drawn from current and prior findings is that attentional capture by environmental stimuli may be an unavoidable experience for individuals higher in trait anxiety regardless of their general level of attentional control and regardless of whether stimuli are threat-related.

Although trait anxiety was not directly related to poorer performance on any of the included behavioral tasks, perceived relative deficits in self-reported attentional control were significantly related to trait anxiety level. Unexpectedly, self-reported less-sensitive attention to neutral environmental stimuli was associated with higher levels of trait anxiety, despite the lack of behavioral deficits. Tortella-Feliu and colleagues [53] suggested that the discrepancy between self-report and behavioral indices of attentional functioning may reflect a tendency among individuals higher on trait anxiety to be self-critical regarding their capabilities despite behavioral evidence of adequate attentional functioning. Not only were both attentional control and attentional sensitivity to neutral environmental information significantly associated with trait anxiety level, we found a reduction in the association between trait anxiety and negative affect after accounting for self-reported attentional functioning. This result suggests that lower extraversion/surgency and perceived poorer attentional functioning may be more salient characteristics of individuals with higher levels of trait anxiety than their perceived level of negative affect.

Although the current study found some evidence of trait anxiety-related sympathetic arousal during the O-Span task, the arousal was not consistent throughout the task, as would be expected if trait anxiety is indeed associated with a need for consistently enhanced effort. Since the expected relation was present only early in the task, it is possible that the initial reactivity is indicative of orienting to the demands of the task. Nevertheless, initial reactivity to the O-Span task moderated the association between early information processing and O-Span performance even when controlling for trait anxiety level. This result is consistent with the interpretation of SCL reactivity being at least a partial index of the cognitive effort expended to compensate for the influence of increased early information processing in order to maintain effective working memory updating. However, this interpretation must be made with caution. Since the current study did not include repeated measurement of state affect, we cannot eliminate the possibility that state anxiety or stress experienced by participants contributed to the SCL reactivity.

### Limitations and Future Directions

The current findings suggest the importance of continued exploration of separable components of dispositional attentional functioning among trait-anxious individuals as well as the balance between these components. Although our findings suggest that hypervigilance associated with trait anxiety may confer an objective benefit to early processing of neutral information, the conditions under which this benefit is present were not explored in the current study. ACT predicts that perceived threat associated with state anxiety or stress increases the influence of stimulus-driven attention on individuals higher in trait anxiety. As such, exploring whether enhanced early processing of neutral information, threat-related information, or both, are present in stressful situations is important to establishing the robustness of this apparent performance benefit of trait anxiety. In addition, future work could also examine whether this moderating effect is shaped by perceived threat. A further increase in the influence of stimulus-driven attention due to induced anxiety should require even greater effort from trait-anxious individuals in order to maintain effective attentional control [1], but the current study did not include a state anxiety manipulation.

Further research could explore not only whether a robust moderation effect exits in threatening situations, but also whether indices of increased effort—be they autonomic, neural, or self-report—have utility in partially explaining affective responses to threat. If enhanced effort does indeed result in significant sympathetic arousal, it may be the case that effortfully maintaining attentional control to compensate for the influence of increased stimulus-driven attention contributes to situational distress. This would be consistent with past work indicating that somatic experience typical of increased sympathetic arousal can be perceived as threat by individuals with elevated levels of anxiety [9]. Experimentally manipulating both state anxiety and task demands on the central executive may help identify the independent and/or interactive influences of cognitive effort and situational stress/threat on affective responding.

Additional research is also needed to explore the mechanisms of discrepancies between behavioral and self-report indices of attentional functioning, as well as whether these discrepancies are of value in understanding how individuals high on trait anxiety respond to daily demands on their cognitive performance. For example, future work might explore whether the extent of discrepancy between self-report and objective indices of attentional performance is associated with a proneness to maladaptive, self-critical thinking or pathological worry.

## 5. Conclusions

The current study provides evidence of the interrelated nature of normative trait anxiety and both behavioral and self-report indices of dispositional attentional functioning. Our findings contribute to an emerging literature suggesting that trait anxiety is associated with better early visual information processing, and that the relation between early visual information processing and attentional control capability varies as a function of trait anxiety level. Additionally, our results indicate that self-reported deficits in attentional functioning are associated with higher trait anxiety, although these perceptions may not align with objective evidence of attentional performance. Overall, the current work underscores the need for continued exploration of objective and subjective indices of attentional functioning associated with trait anxiety. Further research is needed in determining how this disruption is modulated by perceived threat; when, and to what extent, it manifests for particular central executive functions; how it influences cognitive effort; and how it relates to subjective experience. These data will allow us to better understand how individuals across the spectrum of trait anxiety attend to their environments and perceive and react to their own cognitive capabilities.

## Figures and Tables

**Table 1:** Descriptive Statistics for Change Detection and O-Span Performance.

		Accuracy			Response Latency	
Variable	*n*	Mean ^a^	SD	*n*	Mean	SD
Change Detection—Total	55	0.72	0.07	55	760.85	201.79
Small Array—Total	55	0.84	0.10	53	693.35	159.22
Change	55	0.73	0.18	55	746.92	213.61
No Change	55	0.94	0.07	55	691.96	216.83
Medium Array—Total	55	0.70	0.09	55	768.85	202.72
Change	55	0.51	0.17	55	797.50	208.85
No Change	55	0.89	0.08	55	740.20	210.24
Large Array—Total	55	0.62	0.08	55	794.26	220.12
Change	55	0.39	0.15	55	806.96	236.54
No Change	55	0.85	0.10	55	781.57	220.10
Operation Span						
Recall	52	53.35	4.47	--	--	--
Operation Decision	52	0.88	0.09	53	2063.57	350.01

Note. ^a^ Mean value represents total words recalled.

**Table 2:** Moderation by Trait Anxiety of the Relation between the Change Detection Reaction Time and O-Span Recall.

Variable	B	SE(B)	t
Constant	53.000	0.609	87.03 **
Trait Anxiety	0.078	0.048	1.63
CD RT	0.008	0.004	2.14 *
Trait Anxiety × CD RT	−0.001	0.000	−3.28 **
−1 SD Trait Anxiety	0.014	0.004	3.54 **
Mean Trait Anxiety	0.008	0.004	2.13 *
+1 SD Trait Anxiety	0.002	0.004	0.43

Note. ** *p* < 0.01; * *p* < 0.05; CD RT = change detection reaction time.

**Table 3:** Moderation by Initial SCL Reactivity (SCL-R) of the Relation between the Change Detection Reaction Time and O-Span Recall, Controlling for Trait Anxiety.

Variable	B	SE(B)	*t*
Constant	52.66	3.207	16.42 **
SCL-R	0.015	0.017	0.38
CD RT	0.006	0.017	0.89
SCL-R × CD RT	−0.000	0.000	−2.11 *
−1 SD SCL-R	0.010	0.005	2.22 *
Mean SCL-R	0.006	0.006	1.00
+1 SD SCL-R	0.000	0.007	0.09
Trait Anxiety	0.030	0.075	0.40

Note. ** *p* < 0.01; * *p* < 0.05; CD RT = change detection reaction time.

**Table 4:** Descriptive Statistics and Intercorrelations for Trait Anxiety and ATQ Scales.

	Variable	Mean	SD	1	2	3	4
1	Trait Anxiety	37.3	9.59	--			
2	Negative Affect	3.89	0.62	0.441 **	--		
3	Extraversion/Surgency	4.94	0.84	−0.425 **	−0.209	--	
4	Attentional Control	3.76	1.04	−0.338 *	−0.375 **	−0.121	--
5	Neutral Perceptual Sensitivity	4.75	0.82	−0.226	−0.207	−0.029	−0.098

Note. *n* = 56; ** *p* < 0.01; * *p* < 0.05.
